# New Immuno-Epidemiological Biomarker of Human Exposure to *Aedes* Vector Bites: From Concept to Applications †

**DOI:** 10.3390/tropicalmed3030080

**Published:** 2018-08-01

**Authors:** André B. Sagna, Mabo C. Yobo, Emmanuel Elanga Ndille, Franck Remoue

**Affiliations:** 1 MIVEGEC, IRD, CNRS, Univ. Montpellier, BP 64501, 34394 Montpellier, France; andre.sagna@ird.fr (A.B.S.); emmsdille@yahoo.fr (E.E.N.); 2 Institut Pierre Richet (IPR), Institut Nationale de la Santé Publique (INSP), BP 1500 Bouaké, Côte d’Ivoire; 3 UFR Sciences de la Nature, Université Nangui Abrogoua, Abidjan, Côte d’Ivoire, BP 801 Abidjan, Côte d’Ivoire; 4 Department of Medical Entomology, Centre for Research in Infectious Diseases (CRID), P.O. Box 13591, Yaoundé, Cameroun

**Keywords:** *Aedes* exposure, biomarker, arbovirus transmission, salivary proteins, immuno-epidemiology

## Abstract

Arthropod-borne viruses (arboviruses) such as dengue virus (DENV), chikungunya virus (CHIKV), Zika virus (ZIKV), and yellow fever virus (YFV) are the most important ‘emerging pathogens’ because of their geographic spread and their increasing impact on vulnerable human populations. To fight against these arboviruses, vector control strategies (VCS) remain one of the most valuable means. However, their implementation and monitoring are labour intensive and difficult to sustain on large scales, especially when transmission and *Aedes* mosquito densities are low. To increase the efficacy of VCS, current entomological methods should be improved by new complementary tools which measure the risk of arthropod-borne diseases’ transmission. The study of human–*Aedes* immunological relationships can provide new promising serological tools, namely antibody-based biomarkers, allowing to accurately estimate the human–*Aedes* contact and consequently, the risk of transmission of arboviruses and the effectiveness of VCS. This review focuses on studies highlighting the concept, techniques, and methods used to develop and validate specific candidate biomarkers of human exposure to *Aedes* bites. Potential applications of such antibody-based biomarkers of exposure to *Aedes* vector bites in the field of operational research are also discussed.

## 1. Introduction

Mosquitoes are vectors of numerous human pathogens that kill or debilitate millions of humans each year. Those belonging to the genus *Aedes* are vectors of the major viral infections including yellow fever, dengue, chikungunya, and Zika fever [1]. It is estimated that 200,000 new cases of yellow fever occur each year with 30,000 deaths [2], despite the existence of a preventive vaccine. There are an estimated 3.9 billion people at risk of infection with dengue virus (DENV) worldwide [3] and 390 million DENV infections a year, including 500,000 severe forms and over 20,000 deaths [4]. Recent large outbreaks of chikungunya and Zika fever have affected the immunologically naive populations of the Indian and Pacific oceans and of the Americas [5,6,7]. As an example, 693,000 and 500,000 cases of chikungunya and Zika, respectively, were suspected in the Americas in 2016 [8]. All these viruses are transmitted between humans by the bite of infected *Aedes* mosquitoes, mainly *Aedes aegypti* and *Aedes albopictus* [9,10].

The lack and limitations of prophylactic treatment in the vast majority of these diseases forces the use of vector control methods to interrupt transmission. This involves extensive surveillance of these mosquito populations to estimate risk of transmission, to plan vector control strategies, and to assess their impact on the diseases. This surveillance is currently based on entomological methods to identify positive breeding sites, to sample adult mosquitoes by traps, intra-housing spraying, and human landing catches [11]. However, these methods have many limitations in efficiency and accuracy that make them difficult to apply on large scales. Indeed, estimation of immature stage densities is time consuming and expensive with problems of inaccessibility to all potential breeding sites. Furthermore, larval and pupal indicators are focused on immature stages that do not perfectly correlate to the density of adult mosquitoes. The estimation of adult female densities using human landing catches could be more significant for sampling host-seeking mosquitoes and then determining human exposure levels [11,12]. However, due to the infection risk of exposed volunteers to mosquito vector bites, this method evokes obvious ethical concerns that limit its application. Furthermore, entomological methods are mainly applicable at the community level and cannot be used to evaluate the heterogeneity of exposure to mosquito bites between individuals. 

Given all these shortcomings, there is an urgent need for more efficient and sensitive complementary tools to improve the impact of vector control strategies (VCS) and predict the risk of transmission of mosquito-borne diseases, and if possible at both the community and individual levels. The development of such tools needs to explore the close interactions between the human host and *Aedes* mosquitoes. The physical contact between these two protagonists involves physiological interactions through salivary proteins of *Aedes* mosquitoes. Indeed, upon feeding, mosquitoes inject a mixture of bioactive molecules into the host that assist with blood meal acquisition. These molecules include factors that are anticlotting, vasomodulatory, and antiplatelet. Interestingly, some of arthropod salivary proteins are antigenic and initiate an anti-saliva antibody response in the human host [13,14,15,16]. One approach is to quantify the human antibody response to mosquito salivary proteins to define a biomarker of exposure to mosquito bites [17,18,19,20,21,22,23,24]. Indeed, the human antibody response against mosquito salivary gland proteins varies temporally and spatially, and correlates with mosquito density or host biting activity, suggesting the human antibody response can be used to study mosquito–human contact [25]. In addition, biomarkers of mosquito bite exposure can be used to define the ranges of medically important mosquitoes, identify areas of risk for vector-borne diseases, and evaluate vector control interventions.

Even though this approach may appear to be valid, the use of whole saliva or salivary gland extracts (SGE) of *Aedes* vectors as a biomarker of exposure could be inadequate because some salivary factors are shared between mosquitoes or arthropods from different genera that induce immune cross-reaction. In the objective to optimise this promising indicator and to use it on a large scale with high reproducibility, the identification of specific and antigenic salivary proteins or peptides, or both, of *Aedes* has been done. This led to the identification of the putative 34 kDa family secreted salivary protein that appeared to be antigenic and specific to the *Aedes* genus [26]. Interestingly, one peptide (the Nterm-34 kDa) from *Ae. aegypti* saliva was recently identified and validated as a pertinent and specific candidate biomarker of human exposure to bites of major *Aedes* species (*Ae. aegypti* and *Ae. albopictus*) in several countries: Benin, Côte d’Ivoire, Lao PDR, and La Reunion Island [27,28,29,30].

This review focuses on studies highlighting the epidemiological approaches, techniques, and methods used to develop and validate specific candidate biomarkers of human exposure to *Aedes* bites. Potential applications of such antibody-based biomarkers of exposure to *Aedes* vector bites in the field of operational research for arboviral diseases control are highlighted.

## 2. Development of Antibody-Based Biomarker of Exposure to *Aedes* Bites

### 2.1. The Concept

**Figure 1:**
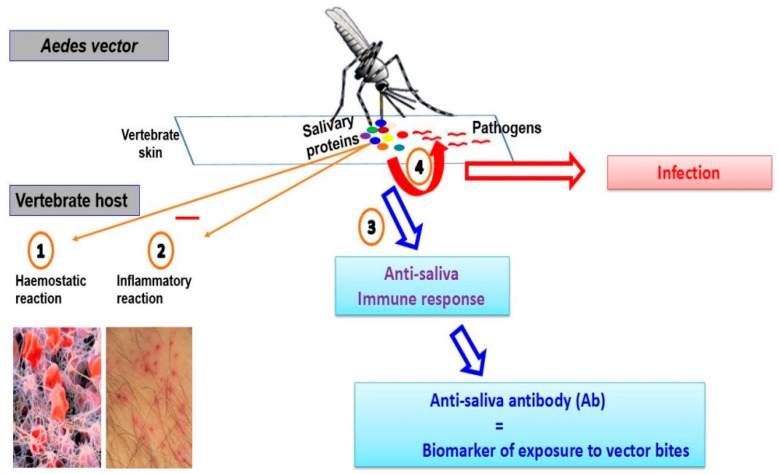
Human-vector relationships during arthropod-borne diseases. During the bite, the vector (*Aedes*) injects its saliva in the human skin. Once in the skin, salivary proteins take the control of (1) the human haemostatic system by inhibiting the platelet activation and clotting mechanism, and (2) the inflammatory system. (3) The salivary proteins modulate the human immune response and promote the production of anti-salivary antibodies. (4) If ever the (*Aedes*) vector carries a pathogen, the salivary proteins contribute to its transmission into the human.

The penetration of haematophagous arthropod mouthparts into the host skin elicits haemostatic, inflammatory, and immune responses by the vertebrate host (animal, human). These reactions aiming to prevent blood loss, promote tissue repair, and prevent colonisation of the damaged tissues by opportunistic pathogens, can largely impair haematophagous arthropod blood intake. Blood-feeding behaviour then requires solutions to counteract vertebrate host defence responses to successfully obtain a blood meal. Thus upon feeding, the female *Aedes*, like all haematophagous arthropods, injects into the mammal host a mixture of bioactive molecules that assist with blood intake by inhibiting physiological responses of the host (Figure 1) [13,31]. Interestingly, some of them (salivary proteins) are antigenic and initiate an antibody response in the host [13,14]. 

The first studies on the effects of mosquito salivary proteins on the immune system of the host concerned allergic reactions due to *Aedes* and *Culex* mosquito bites. These mosquitoes express in their saliva a panel of allergens responsible for a production of specific immunoglobulin (Ig) E antibodies [18,32]. Some of these salivary allergens are now well characterised. For example, four recombinant *Aedes aegypti* salivary allergens corresponding to a 68 kDa salivary apyrase (rAed a1), a 37 kDa protein belonging to the D7 family (rAed a2), a 30 kDa salivary gland allergen (rAed a3), and a 67 kDa α-glucosidase (rAed a4) elicit predominantly specific IgE responses in mosquito-allergic individuals [33,34,35,36]. The authors concluded that these recombinant proteins can be used as tools for diagnosis and treatment of allergic reactions due to mosquito bites.

A panel of serological studies have demonstrated a relation between the exposure of various hematophagous arthropod density and the anti-saliva Ab response levels [21,37]. Regarding *Aedes* mosquitoes, several studies in Finnish Lapland [25], Canada [38], Senegal [17], Bolivia and Reunion Island [23,39], have reported an increasing anti-saliva IgG Ab during the peak of human exposure or in persons living in sites with higher *Aedes* density. Anti-saliva IgG responses have shown to potentially wane just 15 days after a decrease in *Aedes albopictus* densities [40]. Other studies performed in domestic animals showed that IgM antibodies against the saliva of triatomine bugs can be detected after one day of exposure and waned rapidly (18 days) [41,42].

**Figure 2:**
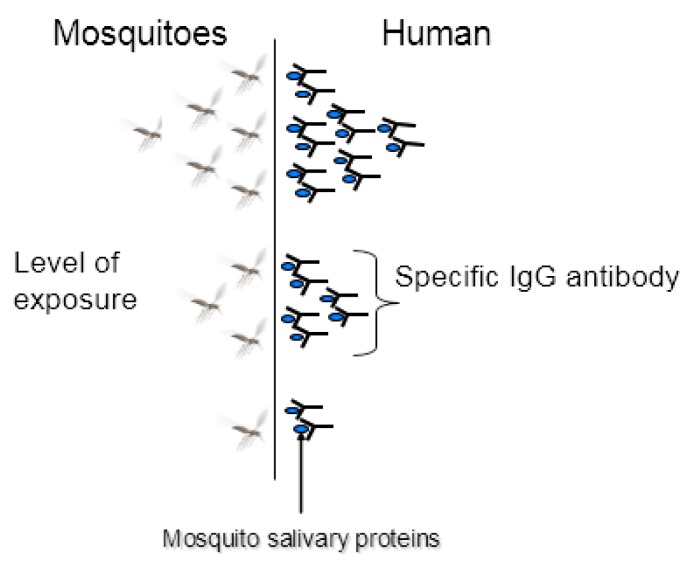
The concept of salivary biomarkers to vector bites.

Since the acquisition of an Ab response against mosquitoes’ (and hematophagous arthropods’) saliva depended on the level of exposure of an individual to mosquito bites, it seemed logical to develop serological tools for monitoring human–vector contacts. The concept ‘anti-saliva Ab response = biomarker of vector bite exposure’ had just emerged. The idea was that the level of specific Ab of an individual is proportional, as a proxy, to the intensity of mosquito bites received by the individual (Figure 2). For example, it has been shown that an individual slightly exposed to *Aedes* bites thus produces a low-level Ab response (≤0.70 ΔOD) to *Aedes* salivary antigens, while a highly-exposed individual to *Aedes* bites produces a high-level Ab response (≥1.2 ΔOD) to *Aedes* salivary antigens [40]. The advantage of such a tool is that it is quantitative (values are expressed in optical densities (OD)) and individual (one OD value represents the level of exposure to bites of one individual).

A low-exposed individual to *Aedes* bites would develop a low-level Ab response to salivary antigens while a highly exposed one would develop a high Ab response to *Aedes* salivary antigens.

### 2.2. Validation of the Concept

To validate the proof of concept ‘anti-saliva Ab response = biomarker of vector bite exposure’, it was necessary to find creative methods to reach this goal. But curiously, all studies undertaken for this purpose were directly carried out in the field. Although this approach could seem surprising, it represents a great benefit for testing the validity of the *Aedes* salivary biomarker as it allows the taking into consideration of specific conditions closely related to the epidemiology of mosquito-borne diseases. To be valid, the *Aedes* salivary biomarker should fulfil four fundamental criteria: (i) to discriminate exposed individuals to *Aedes* vector bites from those who are not exposed, (ii) to evaluate the density and fluctuations of vector populations that can bite human populations, (iii) to classify individuals according to their level of exposure to *Aedes* vectors, and (iv) to be usable at a population level. To demonstrate that the *Aedes* salivary biomarker met these criteria, *Ae. aegypti* SGE were then collected and run on ELISA in serum samples from young Senegalese children living in an area of exposure to the *Ae. aegypti* mosquitoes [20]. Specific IgE and IgG4 responses increased during the rainy season of high exposure to *Aedes* bites, compared to the dry season. In addition, the evolution of anti-saliva isotype levels during the rainy season presented spatial heterogeneity between studied villages. These preliminary results supported the potential approach of using anti-saliva Ab responses for evaluating exposure to *Aedes* vectors and the risk of emerging arboviral infections. 

**Figure 3:**
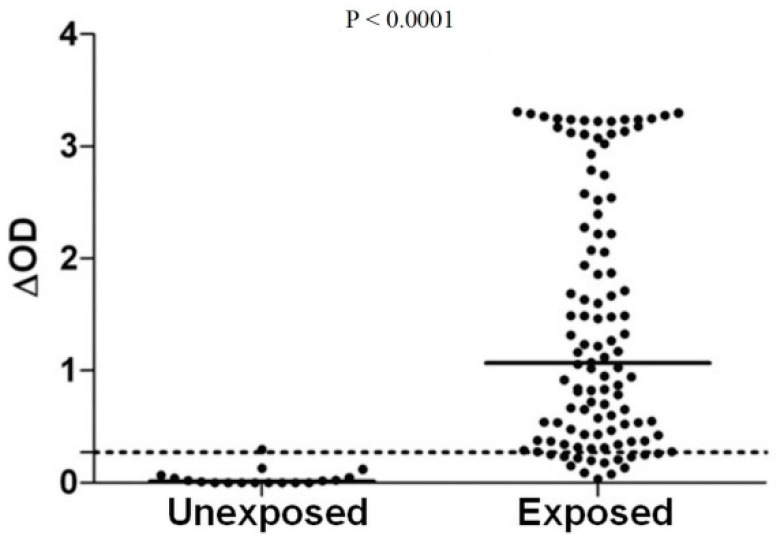
IgG Ab response to *Ae. albopictus* salivary gland extracts (SGEs) between exposed and unexposed individuals to *Ae. albopictus* bites in La Reunion Island [23]. Black points indicate individual IgG response (ΔOD) and bars represent the median value in each group. Dotted line represents the cut-off of specific Ab response (ΔOD > 0.181) and *p* value was calculated using the Mann–Whitney U test.

From these results, several studies were undertaken to evaluate the relevance of using salivary biomarkers to determine the risk of exposure to *Aedes* bites, thus to the viruses that they transmit. For example, several Colombian and French travellers were reported to develop specific Ab responses against *Ae. aegypti* saliva after a stay of 2 to 5 months in areas with *Ae. aegypti* mosquitoes and their specific Ab responses strongly decreased several weeks after the end of their trip [20,24]. In three sites in south-eastern France, a positive association was observed between the average levels of IgG responses against *Ae. caspius* SGE and spatial *Ae. caspius* densities. Additionally, the average level of IgG responses increased significantly during the peak exposure to *Ae. caspius* at each site and returned to baseline four months later [22]. A rapid decrease of anti-*Ae. albopictus* SGE IgG levels was also reported just two weeks after the implementation of control measures (elimination of all breeding sites and insecticide space spraying with deltamethrin) in an urban city in Reunion Island [40]. Other studies using *Ae. aegypti* and *Ae. albopictus* SGE also demonstrated that exposed individuals to these *Aedes* mosquito species developed a high anti-SGE IgG Abs compared to unexposed ones (Figure 3). In addition, anti-SGE IgG Abs were shown to vary according to the level of exposure (measured by entomological methods) and age of individuals [23,39]. 

**Figure 4:**
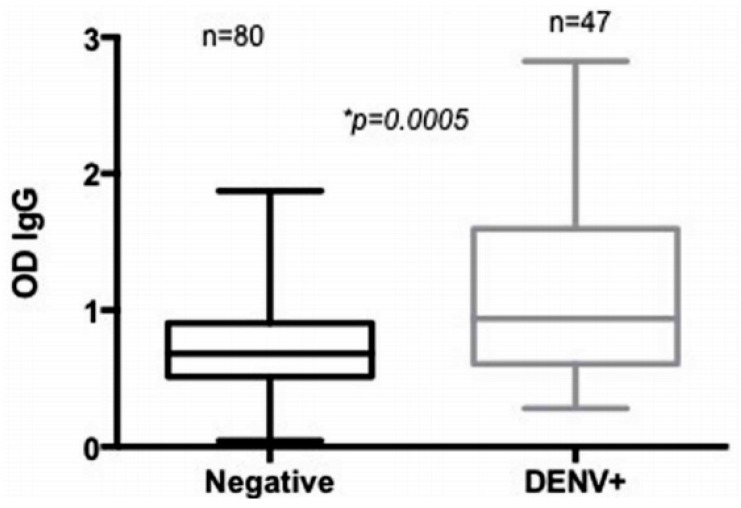
Levels of anti-*Ae. aegypti* SGE antibodies according to the acute dengue infection status in Los Patios in subjects with DENV (dengue virus) (+) (DENV+) and subjects without infection (DENV−) [24]. Statistical significance of Mann–Whitney test * *p* < 0.05.

The relationship between anti-SGE Ab responses and arbovirus infections was also investigated in some studies. Londono-Renteria and colleagues found significantly higher anti-SGE IgG Abs in DENV positive patients with some difference in exposure to *Ae. aegypti* bites among DENV serotypes (Figure 4) [24]. Similarly, the same authors reported higher Ab responses to *Ae. aegypti* SGE in people exposed to DENV, with a positive correlation between anti-SGE IgM and IgG to DENV antigens. In addition, a significant higher level of anti-SGE IgG in subjects living in houses with *Ae. aegypti* aquatic stages was found [43].

Taken together, these results demonstrate that the estimation of human Ab responses to *Aedes* SGEs could be a valuable indicator or a biomarker for evaluating the level of human exposure to *Aedes* bites, the risk of arbovirus infection or transmission, and the effectiveness of vector control strategies. However, a biomarker of exposure based on the use of *Aedes* SGE or whole saliva may not be very reliable for epidemiological purposes. Indeed, saliva is a cocktail of various molecular components with different nature and biological functions, and some of which are shared between species, genus, families, orders, or classes of bloodsucking Diptera or arthropods [13]. Therefore, the evaluation of vector control effectiveness or the risk of arbovirus infection based on the immunogenicity of SGE could be under or overestimated due to possible cross-reactivity between common epitopes. In addition, the collection of saliva or SGEs is tedious and time consuming and saliva batches appeared to be not reproducible in terms of protein quantities and antigenicity. Therefore, it will be difficult or even impossible to have an adequate production of mosquito saliva needed for large scale epidemiological studies. Also, the standardisation of immunological assay using whole saliva appeared difficult and time consuming. An alternative for optimising the specificity of this immunological test would be to identify *Aedes* genus-specific proteins or peptides [44]. 

### 2.3. From the Whole Saliva to Synthetic Salivary Peptide of Aedes

Recent progress in sialotranscriptomic studies have allowed the identification of more specific antigens that have enhanced the specificity of *Aedes* salivary biomarkers [45,46,47]. Two main approaches have been used for that purpose: (i) the recombinant protein strategy based on the identification of genus-specific salivary proteins and (ii) bioinformatics analysis of the sialotranscriptomic data used to design specific and antigenic peptides.

#### 2.3.1. The Recombinant Protein Approach

Proteomic studies on arthropods’ saliva aim to identify which salivary proteins are antigenic and recognised by the vertebrate host during blood intake. These studies revealed the antigenicity of a broad range of arthropod vector salivary proteins [26,48]. This proteomic approach confirmed the Ab cross-reactivity observed in the field with *Aedes* SGE. Western blot analyses on *Aedes* mosquitoes’ saliva revealed common antigens between *Aedes* species. Individuals exposed to *Aedes* bites recognised the 36 and 22 kDa salivary proteins which belong to several *Aedes* species, notably *Ae. communis* and *Ae. aegypti* [18]. Similarly, the screening of *Ae. albopictus* salivary protein using two-dimensional electrophoresis technique combined with immunoblotting and mass spectrometry analysis revealed extensive immune cross-reactivity with *Ae. aegypti* salivary proteins [49]. However, some salivary proteins that may represent candidate biomarkers of exposure at a genus or species level were identified by proteomic tools and produced in their recombinant form so as to be tested under different scenarios. This process led to the development of recombinant proteins such as rTisP14.6 derived from *Triatoma infestans* [50], rLJM17 and rLJM11 proteins produced from *Lutzomyia longipalpis* [51,52], gSG6 from *An. gambiae* [53,54], and fSG6 derived from *An. funestus* [55]. For *Aedes*, one protein, the ‘family secreted salivary putative 34 kDa protein’, was identified and described as specific to the *Aedes* genus [46]. Immunological assays with the recombinant form of this protein in serum samples of rabbits exposed to *Ae. aegypti*, *An. gambiae,* and *Cx. quinquefasciatus* confirmed its antigenicity and its specificity to *Aedes* mosquitoes [56]. Obviously, this protein seemed to constitute a relevant candidate biomarker of exposure to *Aedes* bites. However, the production of recombinant proteins is not an easy task and it is also challenging to produce protein with a high degree of purity and with total reproducibility between production batches to ensure a correct assessment of the anti-saliva Ab response. In addition, recombinant proteins may carry more than one epitope which could increase the risk of immune cross-reactivity, impairing the specificity of candidate biomarkers. To address these limitations linked to the production of recombinant proteins, a peptide approach was developed.

#### 2.3.2. The Peptide Approach

**Figure 5:**
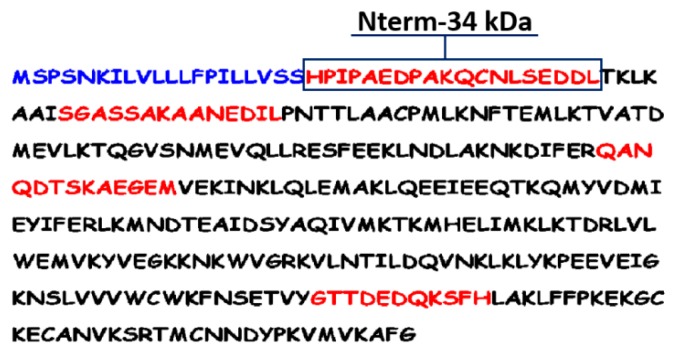
Amino acids’ sequence of the 34 kDa salivary protein of female *Ae. aegypti* mosquito (gi|94468336 [46]). The blue sequence corresponds to the signal peptide.

The peptide approach is based on the exploitation of sialotranscriptome databases and an in silico analysis to identify potential epitopes based on physicochemical properties of amino acids. This peptide design approach was previously used for the identification of the famous and well validated gSG6-P1 (gambiae salivary gland protein-peptide 1) salivary peptide, biomarker of human exposure to major malaria vectors [57,58,59,60]. Basically, several algorithms were employed for prediction of potential immunogenic sites of the putative 34 kDa protein by using bioinformatics. The prediction of immunogenicity was based on the determination of physicochemical properties of the amino-acid (AA) sequences with BcePred and FIMM databases and on the identification of major histocompatibility complex class 2 binding regions using the ProPred-2 online service. This led to defining four peptides (highlighted in red) of 11 to 19 AA residues in length (Figure 5). 

Similarities and specificity were also searched using the Blast family programs, including both the genome and EST libraries of other vector arthropods available in Vectorbase and of pathogens and organisms in non-redundant GenBank CDS databases. All these sequence alignments predicted that only the N-terminal peptide (Nterm-34 kDa) would be very specific to *Ae. aegypti* species and potentially antigenic (Figure 5) [27]. This peptide was then selected and chemically synthetised to be validated as a potential candidate biomarker of human exposure to *Ae. aegypti* mosquito bites. 

#### 2.3.3. Validation of the Synthetic *Aedes* Nterm-34 kDa Salivary Peptide

**Figure 6:**
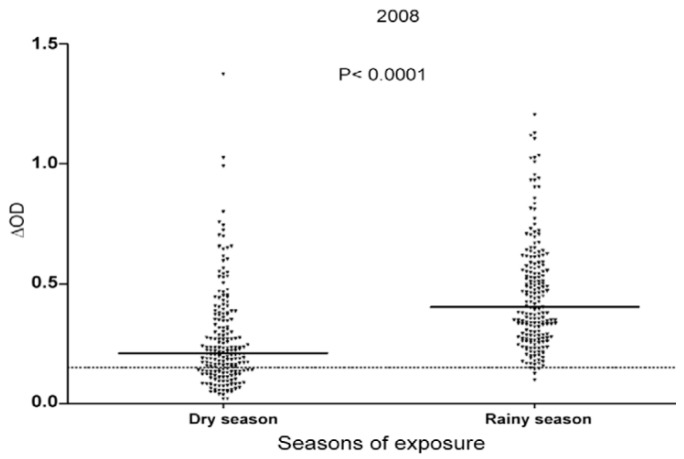
Evolution of individual IgG response to Nterm-34 kDa peptide between the dry and the rainy season [27]. Results are presented for the peak of the dry (February) and the rainy (July) season in 2008. Black points indicate individual IgG response (ΔOD) and bars indicate the median value for each group. Dotted line represents the threshold (TR) of specific Ab response (ΔOD = 0.151) and the statistical significant difference between medians is indicated (non-parametric Wilcoxon test).

The evolution of specific IgG levels to the Nterm-34 kDa salivary peptide was evaluated during dry and rainy seasons in a cohort of 420 under five-year-old children and compared to the accumulated monthly rainfall recorded in the same studied area [27]. A positive association was found between specific IgG Ab responses against the Nterm-34 kDa and the rainfall. Indeed, IgG Ab responses to the Nterm-34 kDa salivary peptide increased significantly from the dry (low exposure to *Aedes*) to the rainy season (high exposure to *Aedes*) (Figure 6). These findings indicate that IgG Ab to Nterm-34 kDa salivary peptide may represent a reliable biomarker to detect the difference, heterogeneity, and evolution in human exposure to *Ae. aegypti* bites, and thus to evaluate the risk of arbovirus transmission. 

This peptide appeared then to satisfy several requirements that an exposure biomarker should fulfil. First, so far, it appears to be specific to *Aedes* genus and therefore, no relevant cross-reactivity phenomena with epitopes from other proteins of arthropods or pathogens would be expected. Second, its synthetic nature guarantees high reproducibility of the immunological assay. Third, it elicits a specific Ab response which correlates well with the level of exposure to *Ae. aegypti* bites. 

Now having a specific biomarker of exposure to *Ae. aegypti* bites, the question is what could be the potential applications of such a tool in epidemiological contexts?

## 3. Potential Applications of *Aedes* Salivary Biomarker in the Field

In endemic areas for mosquito-transmitted diseases like arboviruses, the estimation of the level of human–mosquito contact is an important indicator to measure the risk of transmission of such diseases. For this purpose, discovering that mosquito salivary proteins and peptides can elicit into the human host a specific Ab response that is correlated to its level of exposure to mosquito bites has stimulated researchers to identify specific biomarkers for mosquito bite exposure [17,23,39,57]. Recently, the human IgG Ab response to *Ae. aegypti* Nterm-34 kDa salivary peptide has been validated as biomarker of *Ae. aegypti* and *Ae. albopictus* bite exposure, the main vectors of arboviruses [27]. The availability of such a tool could help to accurately identify individuals exposed to *Aedes* vector bites, consequently at risk of arbovirus transmission, and to evaluate the effectiveness of VCS implemented by sanitary authorities on human–*Aedes* contact.

### 3.1. Evaluation of Human Exposure to Aedes Bites

There is an obvious need of new indicators and methods to evaluate the heterogeneity of human exposure to *Aedes* mosquito bites so as to focus and prioritise interventions in areas, seasons, or groups of individuals at high risk of arbovirus transmission. The relevance of using human IgG Ab responses to the Nterm-34Da peptide as a biomarker of *Aedes* bite exposure was firstly tested in rural areas of Benin, West Africa. Specific IgG Abs to the Nterm-34 Da salivary peptide were evaluated in the same children, during dry and rainy seasons, which generally represented periods of low and high exposure to *Aedes* bites, respectively, and compared with cumulated monthly rainfall registered at the same periods. Specific IgG responses showed significant seasonal variations in the study site. Indeed, specific IgG responses were detected in 29% of children in the dry season, whereas this percentage reached 99% in the rainy season. In addition, the median level of specific IgG Ab responses significantly increased between the dry and the rainy season (Figure 6) [27]. This result perfectly correlated with the intensity of rainfall at the same periods and highlighted the sensitivity and specificity of the Nterm-34 kDa epitope(s). This points out the relevance of using the biomarker of *Aedes* bite exposure for monitoring the risk of arbovirus transmission in areas colonised by *Aedes* mosquito vectors. Recently, another study in urban area of Senegal (Saint-Louis city) revealed considerable individual variations in anti-Nterm-34 kDa IgG levels between and within districts [61]. Similarly, heat maps of IgG responses to the Nterm-34 kDa indicated variations in the spatial distribution of the intensity of Ab responses inside urban districts. Of the four studied urban districts, hot spots of arbovirus transmission risk were located in some patches in the north of one district and were dispersed throughout the other urban district. The two remaining districts showed no hot spots of arbovirus transmission [61]. These results emphasised the significance of using such a serological tool to identify, target, and prioritise vector control strategies in areas with high risk of arbovirus transmission.

So far, studies aiming to validate anti-Nterm-34 kDa IgG Ab as a biomarker of *Aedes* bite exposure were only carried out in areas where *Ae. aegypti* was widely implanted. The study conducted in La Reunion Island revealed a very high immune cross-reactivity between the Nterm-34 kDa peptide of *Ae. aegypti* and that of *Ae. albopictus* [29], the second principal vector of arboviruses worldwide. Indeed, IgG Ab response against the Nterm-34 kDa peptide identified in *Ae. aegypti* saliva was measured in individuals only exposed to *Ae. albopictus* bites. Results indicated that individuals exposed to *Ae. albopictus* recognised the Nterm-34 kDa peptide of *Ae. aegypti*, suggesting that this peptide could be used to evaluate human exposure to these two major arbovirus vectors.

### 3.2. Evaluation of Arboviral Transmission Risk

**Figure 7:**
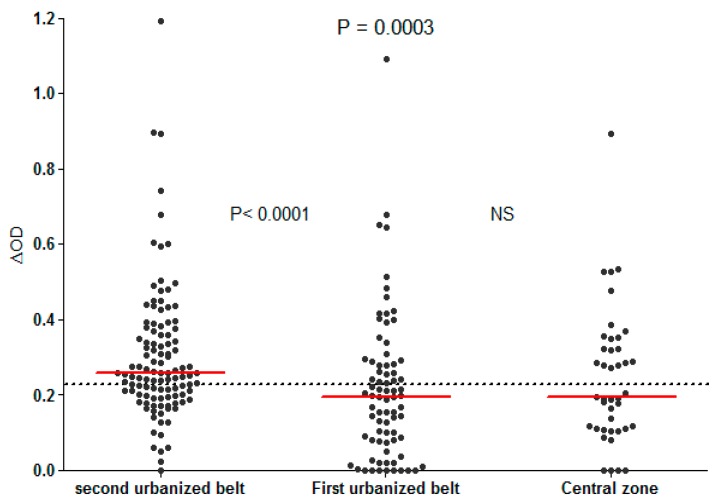
IgG Ab response to Nterm-34 kDa salivary peptide in the studied population according the level of urbanisation of their residential areas in Vientiane city [28]. Black dots indicate individual IgG response (ΔOD), and bars represent the median value in each group. Dotted line represents the cut-off of a specific Ab response (ΔOD > 0.250). Statistically significant differences between the three groups (P = 0.0003, nonparametric Kruskal–Wallis test) and between the first and second urbanised belts (P < 0.0001, Mann–Whitney U test) are indicated.

Previous works provided data on human exposure to *Aedes* saliva and its relationship with dengue infection in the Americas [24,43]. Using the Nterm-34 kDa salivary peptide, one study aimed to evaluate the risk of dengue transmission in urban settings of Vientiane city, Lao PDR [28]. The authors reported no significant difference in anti-Nterm-34 kDa IgG levels between DENV-positive and DENV-negative individuals (evaluated by anti-DENV IgM assays to detect current dengue infection). However, the level of IgG Ab response to Nterm-34 kDa peptide deferred significantly according to the neighbourhoods’ degree of urbanisation (Figure 7). Specific IgG response among individuals living in the second urbanised belt (the periphery with low urbanisation) was higher than those from the first urbanised belt or from the central highly urbanised area. Interestingly, the prevalence of current DENV infection was higher in individuals living in the periphery (4.5%) than those living the central zone (2.2%). This study indicates the potential usefulness of human IgG Ab response to Nterm-34 kDa salivary peptide for predicting areas with a higher risk of dengue virus transmission in urban settings of developing countries. Nonetheless, further studies in different epidemiologic settings are required before validating this biomarker as a proxy of dengue transmission risk.

### 3.3. Evaluation of Vector Control Strategies’ Effectiveness

**Figure 8:**
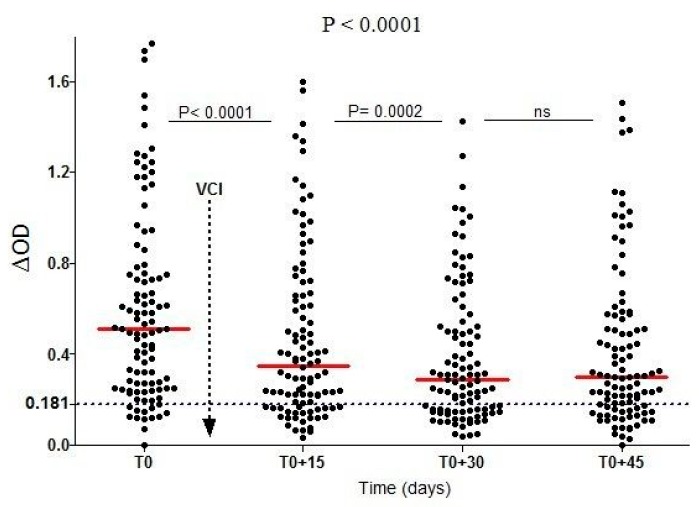
IgG Ab response to Nterm-34 kDa salivary peptide from individuals exposed to *Ae. albopictus* bites before and after vector control implementation [29]. Individual IgG Ab response (ΔOD) is presented just before (T0) and then 15, 30, and 45 days after vector control implementation. Bars indicated the median value in the population at each time point and the dotted line represents the cut-off of immune response. P-values indicating differences in IgG response level at the overall time points (Kruskal–Wallis test) or between two different time points (Wilcoxon matched pair test) are presented. Vertical solid grey line indicates timing of Vector Control Implementation (VCI).

A recent study has reported that human IgG response to the *Ae. aegypti* Nterm-34 kDa peptide could be used for assessing the effectiveness of VCS against *Aedes* [29]. During this study, specific IgG response was assessed from 102 individuals living in urban area in La Reunion Island before and after the implementation of VCS against *Ae. albopictus* mosquito species (elimination of all breeding sites and insecticide space spraying with deltamethrin). A rapid and significant decrease of specific IgG response was reported only 15 days after the VCS implementation. This specific IgG response continued to decrease until 30 days after VCS implementation and then remained unchanged at 45 days (Figure 8). A similar trend was observed with the percentage of immune responders to the peptide. The proportion of immune responders decreased from 88% (before VCS implementation) to 68%, 30 days after VCS implementation. However, a slight increase was observed 45 days after VCS implementation. This decrease in specific IgG response was associated with the decline of *Aedes* mosquito densities, as estimated by entomological parameters and closely correlated to the efficacy of vector control implementation. The impact of implemented VCS were also assessed according the initial level of IgG Ab response in individuals.

All these results showed that this candidate biomarker can detect the short-time variations of human exposure to *Aedes* mosquito bite after vector control implementation. This immuno-epidemiological tool appears to be relevant to assess the effectiveness of VCS against the vectors of arboviruses. 

## 4. Conclusions and Perspectives

This review emphasizes the potential of using biomarkers of human exposure to *Aedes* vectors, based on antibody responses to *Aedes* salivary proteins to evaluate: (i) the level of human population exposure to *Aedes* bites and its heterogeneity, (ii) the risk of arbovirus transmission or infection, and (iii) the effectiveness of vector control means implemented to prevent arbovirus transmission. To improve the specificity of salivary biomarkers, a peptide approach was developed to identify species and genera-specific salivary antigens to *Aedes*. One peptide, the Nterm-34 kDa, was identified as specific to *Aedes* species. IgG Ab to this peptide was validated as biomarker of human exposure to both *Ae. aegypti* and *Ae. albopictus* species. The availability of such a tool allows the assessment of the risk of arbovirus transmission in low-level exposure and transmission areas and to accurately evaluate the effectiveness of current or experimental VCS. For a direct impact in the fight against arboviruses directly in the field and at a large scale, the biomarker is currently in development into a rapid diagnosis test (under its auto-reactive dipstick form) for direct assessment of the level of exposure and the effectiveness of vector control means by sanitary authorities.
